# Presence of sensory nerve corpuscles in the human corpus and cervix uteri during pregnancy and labor as revealed by immunohistochemistry

**DOI:** 10.1186/1477-7827-4-45

**Published:** 2006-08-29

**Authors:** Berith K Tingaker, Gunvor Ekman-Ordeberg, Sture Forsgren

**Affiliations:** 1 Karolinska Institute, Department of Woman and Child Health, Division of Obstetrics and Gynecology, Karolinska Hospital, SE-171 76 Stockholm, Sweden

## Abstract

**Background:**

The uterus is exposed to changes such as enlargement and distension during pregnancy and labor. In these processes and in the process of cervical ripening, proprioceptive information is likely to be of great importance. Therefore, we wanted to study the possible existence of sensory nerve corpuscles in uterine corpus and cervix during pregnancy and labor. Studies on this aspect have not previously been perfomed.

**Methods:**

Biopsies were taken from the upper edge of the hysterotomy during caesarean section at term (n = 8), in labor (n = 5) and from the corresponding area in the non-pregnant uterus after hysterectomy (n = 7). Cervical biopsies were obtained transvaginally from the anterior cervical lip. Serial cryostat sections were prepared for immunohistochemistry using polyclonal antibodies against nerve growth factor receptor p75, protein gene product 9.5 and S-100.

**Results:**

Structures with the characteristics of sensory nerve corpuscles were observed in several specimens after staining for p75, PGP 9.5 and S-100. They were observed in specimens of the non-pregnant corpus and cervix and also in specimens of the pregnant cervix before onset of labor. However, they were absent in all specimens during labor.

**Conclusion:**

Sensory corpuscles have here for the first time been detected in the human corpus and cervix uteri. Studies on the importance of the corpuscles in relation to the protective reflex actions that occur in the uterus during pregnancy should be performed in the future.

## Introduction

The innervation of the corpus and the cervix uteri has been described in several studies focusing on different aspects. Nerve fibers containing sensory neuropeptides such as substance P (SP) and calcitonin gene related peptide (CGRP) are present in the human cervix [1,2] as well as in the cervix of animals [3]. It is also known that marked nerve-related changes occur in the corpus during pregnancy. Studies in both humans [4-6] and animals [7] have shown the occurrence of an almost total denervation of the term pregnant and the laboring corpus. On the other hand, there is still a dense innervation in the cervix throughout pregnancy and labor [3,4,6,8], including sensory innervation [2].

It is likely that nerve-mediated effects play important roles in the processes that occur during labor and cervical ripening, particularly effects involving nerves that transmit afferent proprioceptive information. However, it is not known whether the corpus and cervix uteri are equipped with sensory corpuscles, i.e. structures that might have importance for proprioceptive information. Sensory corpuscules are known to exist in ligaments [9-11], joint capsules [12], skin [13-15] and periodontium [16,17] of various species. Corpuscle-like bodies have also occasionally been observed in the human colon [18]. The corpuscles are known to react on distension and pressure and to play a role as starting points of sensory pathways. It would be of interest to know whether sensory corpuscles also exist in the uterus and convey proprioceptive information during pregnancy and parturition.

The aim of the present study was to explore the possible existence of sensory corpuscles in the uterus. In addition, we wanted to explore possible differences in the pattern of occurrence of corpuscles in the corpus and the cervix, as they have different functions during pregnancy and labor. Biopsies from the corpus and the cervix of non-pregnant and term pregnant women as well as women in labor, were examined by using immunohistochemical procedures.

## Materials and methods

### Patients

Three different groups of patients were studied. One group consisted of seven women who underwent a hysterectomy with a benign diagnosis: six for menorraghia due to fibroids and one for an ovarian cyst. They were all menstruating regularly and none received any hormonal therapy. Another group was comprised of eight term-pregnant women with a normal pregnancy. They had an elective caesarean section performed prior to onset of labor because of fetal breech position, previous complicated vaginal delivery or for psychological reasons. The third group consisted of five term pregnant women in established labor. They had an emergency caesarean section performed due to prolonged labor and/or fetal disproportion. Clinical characteristics of non-pregnant, term-pregnant, and women in labor are given in Table 1.

**Table 1:** Clinical characteristics of non-pregnant, term-pregnant, and women in labor

	Non-pregnant n = 7	Term-pregnant n = 8	In labor n = 5
Mean age (years)	45	33	27
Range	39–50	28–38	21–35
Mean gestational age (completed weeks)		38	40
Range		37–39	37–41
Mean parity	3	1	1
Range	0–4	0–2	0–3
Cervical dilatation (cm)			5
Range			3–10
Mean birth weight (g)		3407	3713
Range		2935–4080	3430–4145

All patients gave their informed consent. The Local Ethics Committee of the Karolinska University Hospital approved the study, which was conducted according to the Declaration of Helsinki.

### Sampling procedure

Biopsies (400–500 mg) were taken from the isthmic part of the corpus uteri. Thus, they were excised from the upper edge of the lower uterine segment, where the incision was made during caesarean section. The abdominal serosa and decidua had been removed. Biopsies from the corresponding area in the non-pregnant uterus were obtained after hysterectomy. The abdominal serosa had been removed but not the endometrium. Cervical biopsies (150–300 mg) were taken transvaginally from the anterior cervical lip, including all layers, at the 12 o'clock position, after caesarean section or hysterectomy.

### Tissue preparation

Tissue specimens were immersed for 20 to 24 h at 4°C in 4% paraformaldehyde in 0.1 M phosphate buffer (pH 7.0). They were thereafter rinsed for 24 h in Tyrode's solution (pH 7.2), containing 10% (w/v) sucrose. The specimens were then mounted on thin cardboard with OCT embedding medium (Miles Laboratories, Naperville, IL, USA) and frozen in propane chilled by liquid N_2 _or in isopentane. Serial cryostat sections of 8 μm in thickness, 15 consecutive sections from each tissue block, were mounted on slides coated with chrome-alun gelatine. They were further processed for immunohistochemical examination, using immunofluorescence or peroxidase antiperoxidase (PAP) stainings, or for demonstration of tissue morphology (hematoxylin-eosin). In order to reveal the sensory corpuscles, stainings for p75 were performed at different positions in the series and adjacent sections were stained for PGP 9.5 and S-100 (see below).

### Immunofluorescence

The sections were incubated for 30 min in a 1% solution of detergent Triton X-100 (Kebo Lab, Stockholm, Sweden) in 0.01 M phosphate-buffered saline (PBS), pH 7.2, containing 0.1 % sodium azide as preservative, and rinsed three times for 5 min each in PBS. The sections were then incubated in normal 5% swine serum in PBS supplemented with 0.1% bovine serum albumin (BSA) for 15 min. They were thereafter incubated with the primary antibody (see under "Antibodies"), diluted in PBS with BSA, in a humid environment. Incubation was performed for 60 min at 37°C. After washing in PBS and another incubation with normal swine serum, the sections were incubated with the secondary antibody corresponding to tetramethylrhodamine isothiocyanate (TRITC)-conjugated swine antirabbit IgG (Dakopatts, Glostrup, Denmark), diluted 1:40, for 60 min at 37°C. They were then washed in PBS and mounted in glycerol:PBS. Examination was carried out in a Leitz Orthoplan photomicroscope equipped with epifluorescence optics. In the case of the staining for PGP 9.5, the sections were initially pretreated with acid potassium permanganate for two min, in order to enhance specific immunoreactions [19].

### PAP-staining

The sections were incubated in a 1% Triton X-100 solution for 20 min. They were then washed in PBS 3 × 5 min and endogenous peroxidase activity was blocked by 30 min incubation in 1% H_2_O_2_. After subsequent washing in PBS, the sections were incubated with normal swine serum for 15 min. Incubation with primary antibody was performed for 60 min at 37°C. After washing in PBS and another incubation with normal swine serum, the secondary antibody was applied (swine anti-rabbit, 1:100, code Z196; Dakopatts, Glostrup, Denmark). The sections were rinsed in PBS 3 times (5 min each), then incubated for 30 min at room temperature with PAP rabbit (1:100, Z0113, Dakopatts), which is prepared from horseradish peroxidase and polyclonal rabbit anti-horseradish peroxidase and to which the secondary antibody used is known to bind. The sections were rinsed in PBS 3 × 5 min once again and developed in diaminobenzidine solution for 5 min. The latter solution was made as follows: one tablet (10 mg) 3,3'-diaminobenzidine tetrahydrochloride (Sigma D-5905) was added to 10 ml 0.05 M Tris buffer and 100 μl 1 M imidazole, to which 10 μl 30% H_2_O_2 _was also added. After the sections had been incubated in the diaminobenzidine solution, they were dehydrated and finally mounted in DPX microscopy mounting medium. In the case of staining for PGP 9.5, initial treatment with acid potassium permanganate was used (cf above).

### Basis for identification of sensory corpuscles

Several types of staining were used to identify sensory corpuscles. Staining for the nerve growth factor receptor p75 was done at several levels in the series of sections that were cut. This staining has frequently been utilized in previous studies to characterize these corpuscles [11,20,21]. Other sections in the series were processed for protein gene-product 9.5 (PGP 9.5) and S-100 protein. PGP 9.5 staining was used to label the central axon of the sensory corpuscle and S-100 was used for the delineation of the periaxonic Schwann cells of the inner core of the corpuscles [10,15].

The sections were examined under a Zeiss Axioplan II microscope equipped with an Olympus DP10 digital camera.

### Antibodies

Rabbit antibodies against PGP 9.5 (Biogenesis, Poole, England; code 7863–0504; working dilution 1:500), S-100 (Sigma, St. Louis, MO, USA; code S2644; working dilution 1:50) and the low-affinity p75 (Sigma, code N-3908; working dilution 1:100) were applied. According to the supplier, the antibody against PGP 9.5 reacts with PGP 9.5 in all mammalian species tested, including humans. The S-100 antibody specifically stains the S-100 protein, including in the Schwann cells of the peripheral nervous system. The p75 antibody is described by the supplier as reacting specifically with NGF receptor p75 (75 kd), and staining the NGF receptor p75 band when used for immunoblotting. It is reported to be specifically inhibited by an immunizing peptide (rat NGF receptor, amino acids 407–425 with N-terminally added lysine). In control experiments, the primary antibodies were omitted or replaced with normal serum. As additional controls, incubation with antibodies against PGP 9.5, p75 and S-100 was performed on slides of tissue known to contain nerve structures including sensory corpuscles (wrist ligaments) [21].

## Results

### Presence of sensory corpuscles

Structures were identified in the non-pregnant corpus and the non-pregnant cervix as well as the term-pregnant cervix (before onset of labor), which on the basis of their patterns of immunoreactivity for p75, PGP 9.5 and S-100 (cf below) were classified as sensory corpuscles. Their morphologic appearances clearly distinguished them from nerve fascicles in the tissue.

**Figure 1:**
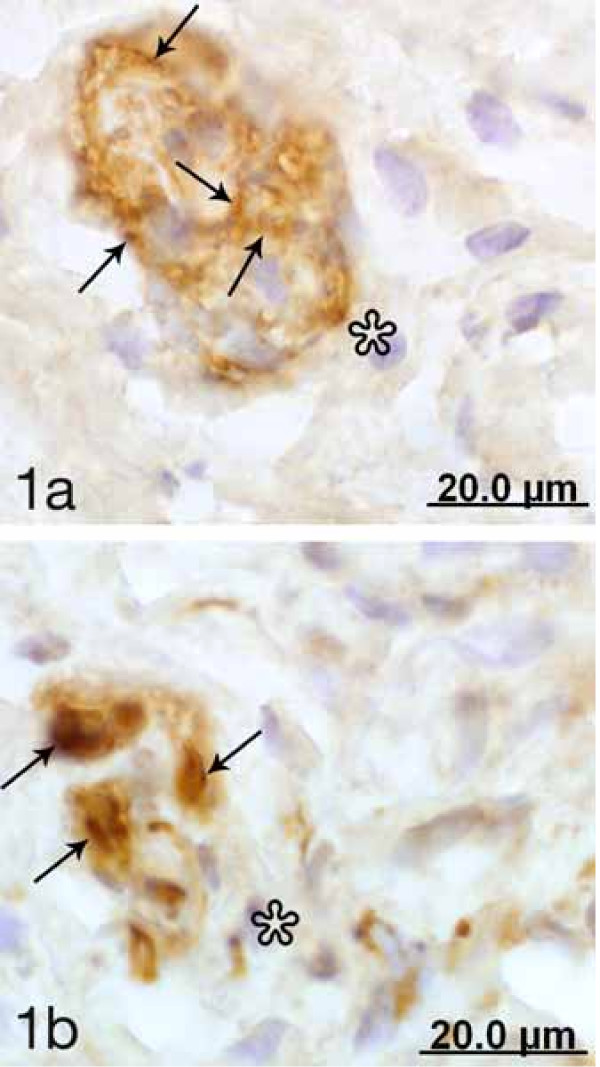
Two sections in the series of sections of a corpuscle and adjacent tissue from a non-pregnant cervix stained for p75 (a) and PGP 9.5 (PAP-staining) (b). Asterisks at corresponding regions just outside the corpuscle. PGP 9.5 immunoreactive nerve fibers are seen within the corpuscle (black arrows). The occurrence of intracorpuscular nerve fibers is observed via PAP staining (arrows, b). Compartmentalization is evident in (a); p75 immunoreaction delineates the contours of the compartments (arrows).

**Figure 2:**
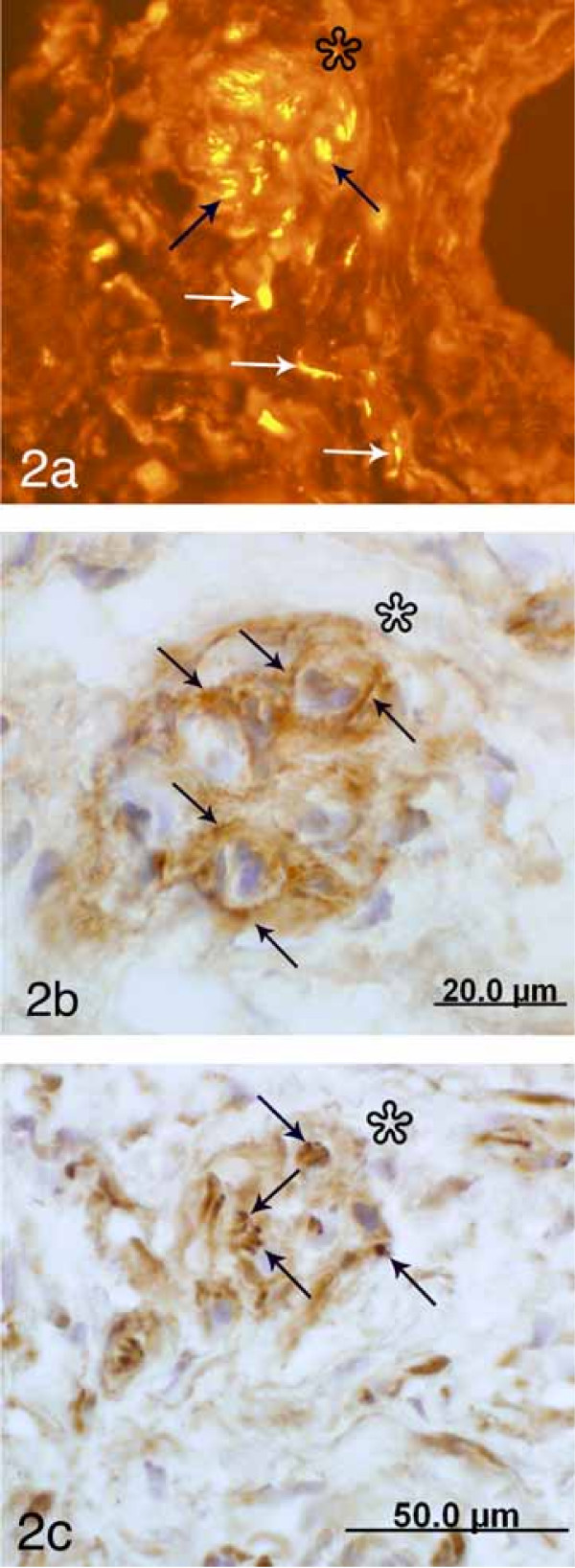
Sections from the serial sectioning of a specimen from non-pregnant cervix showing a corpuscle and surrounding tissue. The sections were processed for PGP 9.5 (TRITC staining) (a), p75 (b) and PGP 9.5 (PAP staining) (c). Asterisks at corresponding regions just outside the corpuscle. The corpuscle and the surrounding tissue are shown at lower magnification in (a) than in (b-c). Part of a blood vessel is visible to the right in (a). Arrows in (b) indicate p75 immunoreaction at the peripheries of the small structures that comprise the corpuscle. Black arrows in (a and c) indicate nerve fibers occurring within these small structures. There is a marked immunofluorescence reaction in (a) not only in these nerve fibers but also in approaching nerve fibers (white arrows).

**Figure 3:**
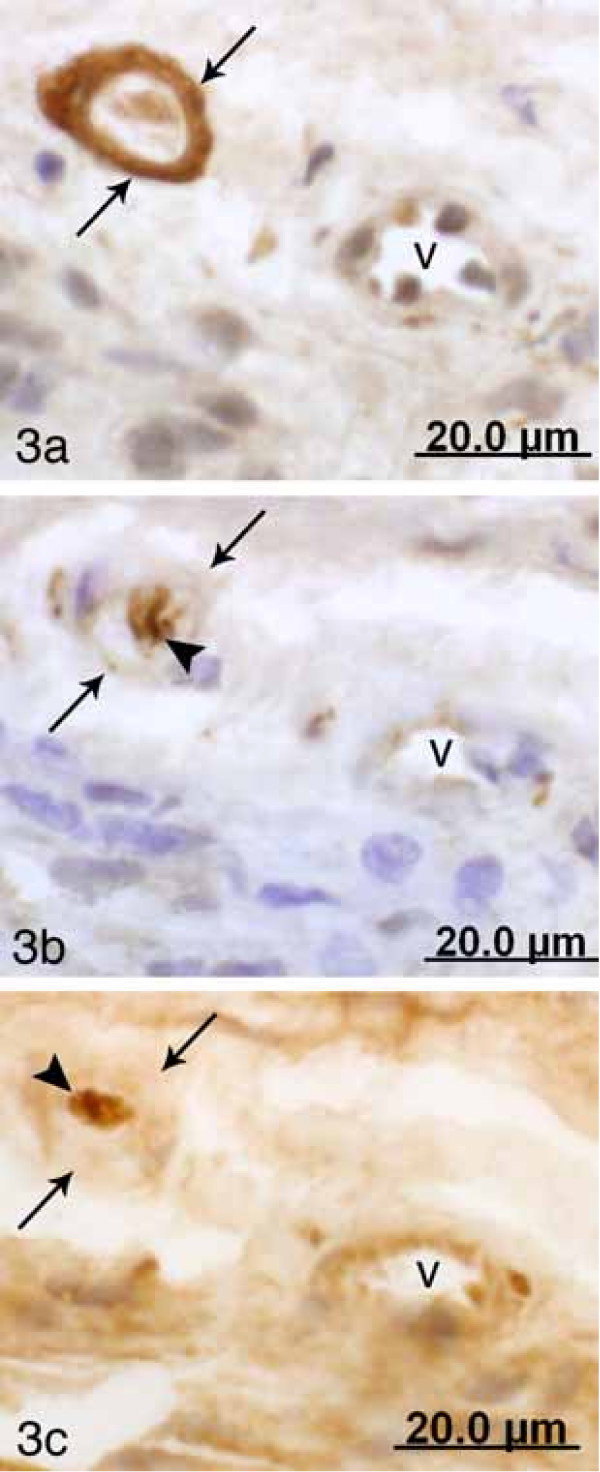
Parallel sections from term pregnant cervix processed for p75 (a) PGP 9.5 (b) and S-100 (c). A small corpuscle with marked capsular p75 immunoreactivity is seen in (a, arrows at capsule). Corresponding capsular regions in the PGP 9.5 (b) and S-100 (c) stained sections are also marked with arrows. Fine PGP 9.5 immunoreactive nerve fibers are observed within the corpuscle (b, arrowhead). S-100 immunoreaction is also observed in the interior of the corpuscle (c, arrowhead). V (a-c) at a nearby located transversely cut blood vessel.

Some of the sensory corpuscles observed in the present study had a well-developed capsule, showing a distinct p75 immunoreaction, whereas others did not have a clearly defined capsule and showed a diffuse p75 immunoreaction (Fig 1a, 2b, 3a). Serial sections revealed that the sensory corpuscles were rounded or oval structures with a diameter of 25–75 μm, often located in the vicinity of blood vessels (Fig 2, 3).

There was a lack of PGP 9.5 and S-100 immunoreactivity in the capsule of the corpuscles. On the other hand, PGP 9.5 immunoreactive nerve fibers (Fig 1b, 2a and 2c, 3b) as well as S-100 immunoreactions (Fig 3c) could be seen within the structures. The occurrence of PGP 9.5 immunoreactive nerve fibers was revealed by PAP staining (cf Fig 1b, 2c, 3b) and particularly by TRITC staining (cf Fig 2a). Small nerve fascicles containing nerve fibers showing PGP 9.5 immunoreaction were seen approaching the corpusles (Fig 2a).

The corpuscles showing a diffuse p75 immunoreaction in their capsule were often seen to harbor small intra-capsular corpuscular structures exhibiting a clearly defined p75 immunoreaction in their peripheries (Fig 1a, 2b). Thus, these corpuscles showed a compartmentalized appearance. PGP 9.5 immunoreactive nerve fibers occurred in the interiors of these small corpuscular structures (Fig 3c). The occurrence of this compartmentalization further aided in the distinguishing of corpuscles.

### Frequency of sensory corpuscles

Sensory corpuscles were clearly identifiable in the non-pregnant stage in five out of seven cervical specimens (cf Fig 1, 2) and in four out of seven specimens of the corpus. In the term-pregnant cervix, clearly identifiable corpuscles were distinguished in three cases out of eight (cf Fig 3). They were not detected in any of the specimens of the cervix during labor. They were not observed in the term-pregnant corpus nor in the corpus during labor (Table 2).

**Table 2:** Presence of sensory corpuscles

	Non-pregnant	Term-pregnant	In labor
Corpus	+ (4/7)	- (0)	- (0)
Cervix	+ (5/7)	+ (3/8)	- (0)

### Control stainings

There was no specific staining for p75, PGP 9.5 and S-100 in the control sections from the corpus and the cervix. Parallel examination of sections of control specimens (ligament structures) aided in the identification of the sensory corpuscles.

## Discussion

Structures we interpret as being sensory corpuscles were identified in the present study. They were seen in both the non-pregnant corpus and the non-pregnant cervix, as well as in the term-pregnant cervix before onset of labor. The corpuscles were identified via parallel stainings for p75, S-100 and PGP 9.5. Sensory corpuscles in the human uterus have here been described for the first time.

From previous studies it is known that the capsule of sensory corpuscles is an expansion of the perineurium [13], [22]. In the present study, the capsule was found to be well-defined, showing distinct p75 immunoreactivity that ranged from diffuse to distinct. These observations correspond to observations made for corpuscles in other locations [16,20]. The finding that the central regions of the corpuscles showed S-100 immunoreaction are in accordance with the observations that the Schwann-related cells in the center of corpuscles in the skin [23], the posterior cruciate ligament [10] and the scapholunate interossus ligament [11] of humans express the S-100 protein. It is well known that the central arborizing axons of the corpuscles, which correspond to the peripheral processes of Aα, Aβ and Aδ sensory axons, are surrounded by Schwann-related cells. The axons of the corpuscles, including those within the small corpuscular structures, exhibited marked PGP 9.5 immunoreaction.

Sensory corpuscles are classified in different ways in the literature, and even within a certain classified type the appearance can vary [13]. Nevertheless, the corpuscles that were found to be distinctly encapsulated in the present study, with a distinct capsular p75 immunoreaction, closely resemble small Pacini-corpuscles [24-26]. On the other hand, the corpuscles that had an incomplete capsule, with an indistinct capsular p75 immunoreaction, to a certain extent resemble the Ruffini type of corpuscles [22], where the perineurium of the supplying nerve fascicle creates an incomplete perineurial capsule around the corpuscles [26].

We found that some corpuscles showing an indistinct p75 immunoreaction at the outer capsule were comprised of small corpuscular structures exhibiting p75 immunoreaction in their peripheries and having PGP 9.5-immunoreactive axons in their interiors. We have recently made similar observations for corpuscles in the human wrist ligaments; these were interpreted as being Ruffini type corpuscles [21]. The small corpuscular structures seen in the present study were arranged in cylinders, as described in studies on sensory corpuscles in the cruciate ligaments [22]. The peripheries of the cylinders are formed from perineurial cells that surround the afferent axons. Previous studies have revealed that the axon branches into enlarged nerve terminals. These are anchored between bundles of collagen fibers inside the cylinders [22]. Accordingly, the perineurial cells of the small corpuscular structures were found to exhibit p75 immunoreaction and PGP 9.5 immunoreactive nerve fibers. The nerve fibers were found in the interior of the cylinders. Future studies at the ultrastructural level will further clarify the nature of the corpuscles.

Sensory corpuscles were detected in the non-pregnant corpus. Interestingly, they were not observed in the term-pregnant corpus nor during labor. The observations should be interpreted with caution, as corpuscles were not seen in all specimens of non-pregnant corpus and as the specimens analyzed cannot be said to be fully representative of the different stages. For comparison, nerve fibers almost completely disappear from the corpus during late pregnancy and labor [4-7]. From previous studies it is known that corpuscles on the whole may be dependent on the trophic influence of sensory axonal afferents [27,28]. Thus, denervation leads to degeneration of sensory corpuscles [29,30]. Whether there is a causal relationship between the disappearance of corpuscles and the well-known diminution of the innervation and/or the very marked structural rearrangement including the remodeling of the extracellular matrix of the corpus should be examined in further studies.

We found corpuscles in the cervix uteri of the non-pregnant group and also, in contrast to the corpus, in that of term-pregnant women. However, corpuscles were not observed in the cervix during labor. For comparison, the general cervical innervation is dense throughout pregnancy [3,4,6,8].

It has been suggested that the density of sensory corpuscles in ligaments may be related to the occurrence of protective reflexes during extreme movements [31]. The concentration of small Pacinian corpuscles to various regions of joints is discussed in terms of protective and stabilizing actions [9]. To what extent the sensory corpuscles in the corpus and the cervix uteri also may have a relation to protective reflex actions should be further examined. It may be postulated that both the presence and the activity of sensory corpuscles are influenced by pressure, distension and displacement. In the non-pregnant stage, the corpus, which mainly consists of smooth muscle [32], responds to distension and pressure with pain and small contractions, trying to maintain its size. An interesting aspect that should be considered in future studies is the possibility that the sensory corpuscles play a role in this protective action. During pregnancy the cervix remains closed until the final softening, recognized as remodeling of the extracellular matrix. It is appealing to speculate that sensory corpuscles participate in that process, for instance contributing to the prevention of premature cervical softening. It is noteworthy that corpuscles were observed in only half of the samples from term-pregnant cervix. However cervix at term can be at different stages of ripening. Maybe the ripening process had started in some of the samples and affected the presence of corpuscles. Further investigations are needed to clarify the function of uterine sensory corpuscles.

## Conclusion

Structures interpreted as sensory nerve corpuscles in the human corpus and the cervix uteri have here for the first time been described. Thus, the uterus can be added to the list of organs containing these structures. It is well known that the corpus and the cervix serve different functions during pregnancy. The corpus distends in order to host the growing fetus. In contrast, the cervix remains closed in spite of the increasing pressure, until final cervical ripening and onset of labor. Presumably, the corpuscles have roles in these processes. Further studies are needed in order to clarify the details in the classification and the roles of the corpuscles.

## Abbreviations

BSA bovine serum albumin

p75 nerve growth factor receptor p75

PAP peroxidase anti peroxidase

PBS phosfate-buffered saline

PGP 9.5 protein gene product 9.5

TRITC tetramethylrhodamine isothiocyanate

## Authors' contributions

B. K Tingåker has selected and recruited the patients, collected all the biopsies, participated in designing the study, performed parts of the immunohistochemistry, evaluated the specimens and drafted the manuscript. G. Ekman-Ordeberg participated in the study design, analysis and discussion of the results and critical revision the manuscript. S. Forsgren supervised the immunohistochemistry, evaluated the specimens, participated in the study design, analysis and discussion of the results, drafting and critical revision of the manuscript. All authors read and approved the final manuscript.
